# Identification of soil particle size distribution in different sedimentary environments at river basin scale by fractal dimension

**DOI:** 10.1038/s41598-022-15141-6

**Published:** 2022-06-29

**Authors:** Yanyan Wang, Yujiang He, Jiang Zhan, Zhiping Li

**Affiliations:** 1 College of Geosciences and Engineering, North China University of Water Resources and Electric Power, Zhengzhou, 450045 China; 2 The Institute of Hydrogeology and Environmental Geology, Chinese Academy of Geological Science, Shijiazhuang, 050061 China; 3 Yellow River Engineering Consulting Co. Ltd., Zhengzhou, 450003 China; 4 Collaborative Innovation Center for Efficient Utilization of Water Resources, Zhengzhou, 450045 Henan Province China

**Keywords:** Ecology, Environmental sciences, Hydrology

## Abstract

The geomorphology of river basin is complex, and its soil sedimentary characteristics are poorly defined. To study the spatial variability of soil structure in different sedimentary environments at the basin scale, 356 sets of soil samples were collected from five typical sedimentary environments in the Yellow River Basin and the Haihe River Basin, including the upper and lower reaches of the rivers, mountain-front plains, central alluvial plains and eastern coastal plains. The particle size distribution (PSD) of the soil samples was obtained using a laser particle size analyzer, and the fractal dimension (D) of the soil structure was derived by applying fractal theory. The PSD, D and the correlation between them were analyzed by  the Pearson correlation method for typical sedimentary environments in two basins. The results show that: (1) The main soil types in the typical geological environments in the basin are sand, loamy sand, sandy loam, silty loam, and silty soil. The soil particle size in the upper and lower reaches of the rivers was higher than that in the plain areas. (2) In the plane, The D value descended in different regions in the following order: the mountain-front plain > the eastern coastal plain > the upper Yellow River > the central alluvial plain > the lower Yellow River. In the vertical direction for both rivers, the D value showed a decreasing trend with increasing burial depth. (3) The model results showed a cubic polynomial correlation between D values and PSD, which was closely related to the non-uniformity of particle size during sorting and deposition. The soil PSD and fractal characteristics are effective tools for the quantitative evaluation of soil structure in various sedimentary environments in the basin.

## Introduction

River basins are important ecosystems, and their abundant water resources are important for human life, agricultural irrigation, and decontamination^1,2^. However, due to the destruction of ecological environments by human beings and the over-exploitation of resources, environmental problems, such as declining self-cleansing capacity of river basins and degradation of ecological functions, are becoming increasingly important^3–5^. The basin has complex landforms and diverse causes, forming various types of landforms such as plains, deserts, Loess Plateau, hills, and mountains^6,7^. Different depositional environments have influenced soil structure and changed soil physical properties^8^. Soil particle size distribution (PSD) is an important indicator in sedimentological studies, and its characteristics often reflect different depositional dynamics, depositional environment, and source characteristics^9–11^. Thus, understanding the PSD characteristics of typical depositional environments is essential for ecological restoration and soil rehabilitation in the basin.

Particle size distribution (PSD) indicates the relative proportion of different particle sizes in the soil that affect other soil properties^12–14^. Changes in soil PSD can be used to indicate soil compaction and formation processes, and can accurately estimate the hydraulic properties of soils^15–17^. Therefore, it is crucial to find key techniques to quantitatively study PSD characteristics^18,19^. Since Tyler and Wheatcraf^20^ introduced fractal theory into soil science research, it has been widely used in the study of soil particle size characteristics. Fractal characteristics can describe the overall characteristics of the soil PSD, as well as the coarseness of the soil particles^21–23^.

The PSD distribution characteristics of soil and its spatial variation (grain size trend) are mainly controlled by multiple factors such as source components, transport mode, transport distance, hydrodynamic conditions, and topography^24–26^. Sediment composition and grain size characteristics have obvious advantages in the study of sedimentary environment zoning in offshore, estuary, and delta areas^27–29^. In recent years, scholars at home and abroad have made many systematic research results on sediment types in typical geomorphic regions. Wei et al.^30^ discovered that the fractal dimension D_m_ of the Loess Plateau Zone is related to the trend of soil PSD in the adjacent sediment layers, and pointed out that the fractal dimension is an effective parameter for evaluating landuse types and soil degradation processes in typical landform types. Wang et al.^31^ analyzed the spatial distribution characteristics of the surface sediment particle size composition and particle size parameters in different sections of the Qiantang Estuary, and the spatial distribution of particle size characteristics in the study area is in good agreement with the zoning of the dynamical sedimentary environment. Wided et al.^32^ found that the cause of deposition was due to intense wind erosion through soil grain size characterization. The Yellow River Basin and the Haihe River Basin are in important agricultural production areas of China, which are the main economic zones of China^33–36^. However, few studies have been conducted on the variability of soil physical properties in different sedimentary environments, such as river scouring and deposition in the basin.

The objectives of this study were to (1) evaluate the variability of soil particle coarseness and particle size distribution under various sedimentary environments in the basin, and (2) compare the physical properties of soils with fractal characteristics to explore the significant influencing factors of soil particle size fractal characteristics. This was done to provide soil particle size and fractal indicators for the quantitative evaluation of soil texture and soil particle loss in different sedimentary environments in the watershed.

## Materials and methods

### Study area

**Figure 1:**
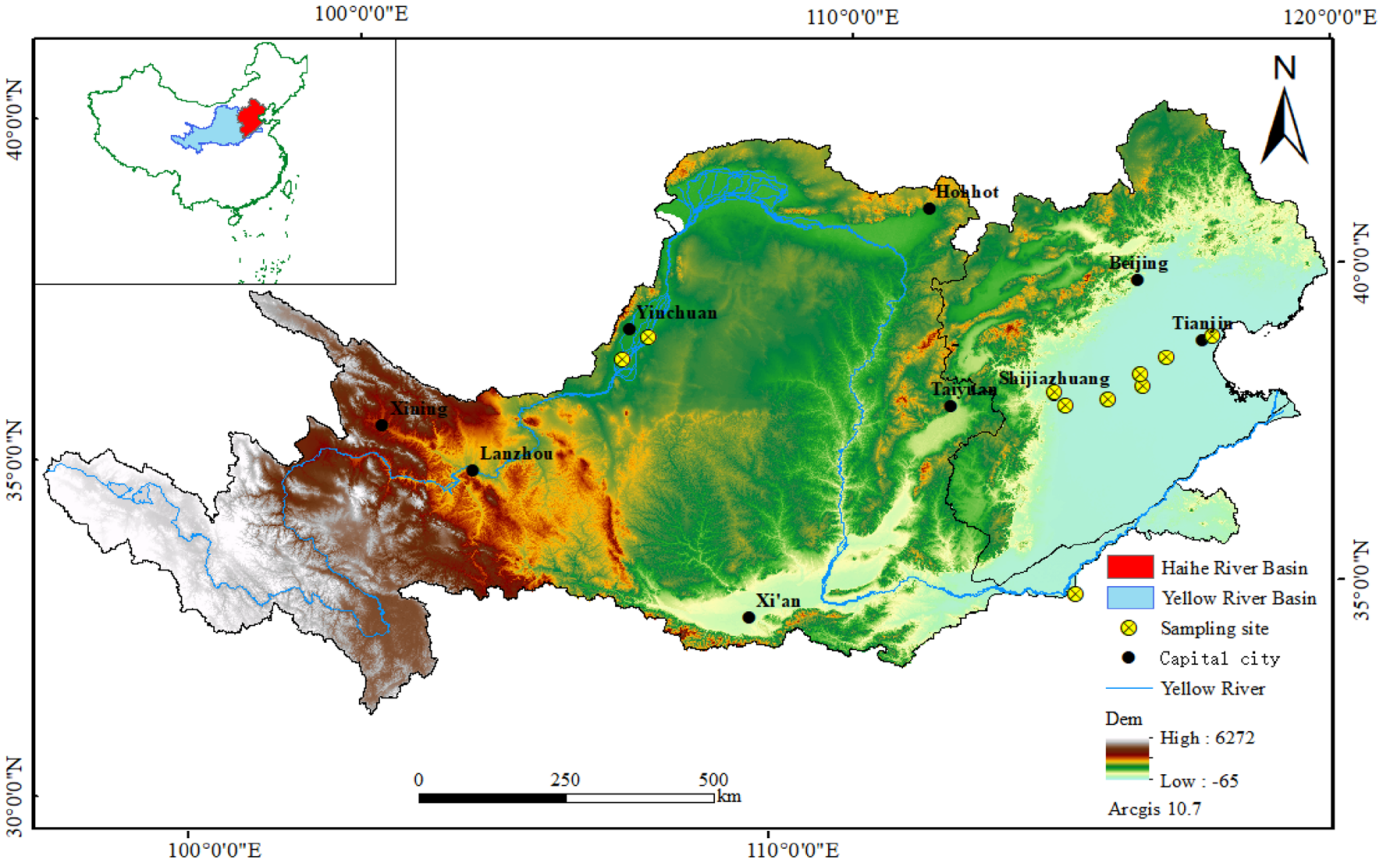
Schematic map of the study area showing the location of soil sampling sites.

The Yellow River Basin has a fragile natural ecology, a shortage of water resources, and distinctive regional differences in endowments of land, mineral, biological, and other resources^37^. It is located between 95°53–119°05E, 32°10–41°50 N; it is 1900 km long from east to west and 1100 km wide from north to south, with a basin area of 795,000 km^2^. After the fourth major diversion of the Yellow River, the Yellow River channel is far away from the lake area, and the lake gradually silted up and died out. The upstream precipitation is long-lasting and has a low intensity, forming small peaks in runoff and large amounts of flood runoff. On the banks of the Yellow River, from Haheyan to Hekou Town, is the Ningmeng Irrigation Area, which is an important agricultural center in the Yellow River Basin. Soil erosion in the Loess Plateau is serious, with an erosion area of 45.4 km^2^. A large amount of sediment is imported into the Yellow River; this silts up the lower reaches of the riverbed, resulting in serious floods in the lower reaches that are difficult to control. The surface sediments of the Yellow River are terrigenous debris, and there are residual sediments in some areas^38^. From shore to sea, sediments are distributed in bands from coarse to fine. The coastal area is dominated by fine sand, interspersed with gravel and other coarse clastic material. The west is the early input of the Yellow River and the Yangtze River. The deep-water area in the central part of China is fine sediment dominated by argillaceous materials, mainly from the Yellow River. The study area is shown in Fig. 1.

The Haihe River Basin is located between 112°–120°E and 35°–43°N, bordered by the Shanxi Plateau and the Yellow River Basin in the west, the Mongolian Plateau and the Inland River Basin in the north, the Yellow River in the south, and the Bohai Sea to the east. It is one of the areas in China with serious floods, waterlogging, drought, and alkali disasters^39^. The basin spans eight provinces, including Beijing, Tianjin, Hebei, Shanxi, Henan, Shandong, Inner Mongolia, and Liaoning, with an area of 320,000 km^2^, accounting for 3.3% of the total area of the country. From east to west, the basin is composed of coastal plains, inland plains, mountainous areas, and plateau landforms. The terrain is high in the northwest and low in the southeast, the plain area in east and south, Shanxi plateau and Taihang mountain area in the west, Mongolia plateau and Yanshan mountain area in the north. There are two types of river systems, one is originated from the leeward slope of Taihang Mountains and Yanshan Mountains, with concentrated tributaries, large catchment area, and high sediment content; the other category originates from the windward slope of Taihang Mountain and Yanshan Mountain, with dispersed tributaries and staggered distribution between the two types of rivers.

### Soil sample collection

In this study, soil structures were studied in five regions with varying drivers of deposition, namely, the upper and lower reaches of waterways in the Yellow River Basin, the mountain-front plains, the central alluvial plains, and the coastal plains in the Haihe River Basin plain area. The PSD characteristics and fractal dimensions of 356 groups of soil samples were analyzed using the fractal method while considering other significant factors influencing the fractal characteristics of soil particle size. Soil PSD and fractal characteristics were used as indicators to reflect the changing characteristics of soil structure under different geological environments. The data used in this experiment were obtained by drilling samples from typical sedimentary environments in the basin, involving different geomorphological features such as mountain-front plains, central alluvial plains, coastal plains, and upstream and downstream rivers. A total of 356 groups of samples were collected. The selected sampling plots and collected samples highlight the differences in soil particle composition; the soil particle compositions were mainly influenced by different geological environments, such as river erosion and deposition.

A total of 210 groups of soil samples were collected from the Haihe River Basin, including 60 groups from the mountain-front plain (distributed in Zhengding County and Luancheng District, Shijiazhuang City), 90 groups from the central alluvial plain (distributed in Xian County, Cangzhou City, and Shenzhou County, Hengshui City), and 60 groups from the eastern coastal plain (distributed in Dacheng County, Langfang City, and Binhai new-region, Tianjin City). The sampling depth was 0–3 m, and samples were collected every 10 cm. The distribution of sampling points and the number of samples are shown in Table 1.

**Table 1:** The description of sampling points.

	Sample num.	Depth (m)	Location	District	Geographical coordinates	
					ψ (N)	λ (E)
Yellow river basin	YS01-30	0–3.0	Yesheng Town, Qingtongxia County, Ningxia Province	Upper yellow river	106°06′45″	38°07′06″
	HL01-30	0–3.0	Helan County, Yinchuan City, Ningxia Province		106°33′03″	38°30′42″
	LK01-86	0–5.0	Lankao County, Kaifeng City, Henan Province	Lower yellow river	114°57′23″	34°54′27″
Haihe river basin	SJZ01-30	0–3.0	Shijiazhuang City, Hebei Province	Mountain-front plain	114°28′33″	38°04′59″
	LC01-26	0–3.0	Luancheng County, Shijiazhuang City, Hebei Province		114°40′58″	37°53′16″
	SZ01-30	0–3.0	Shenzhou County, Hengshui City, Hebei Province	Central alluvial plain	115°30′58″	37°59′14″
	XX01-30	0–3.0	Xianxian County, Cangzhou City, Hebei Province		116°10′11″	38°12′29″
	HJ01-30	0–3.0	Hejian County, Cangzhou City, Hebei Province		116°07′55″	38°23′53″
	DC01-30	0–3.0	Dacheng County, Langfang City, Hebei Province	Eastern coastal plain	116°38′20″	38°39′37″
	BH01-30	0–3.0	Binhai new-region, Tianjin City		117°32′24″	39°00′48″

A total of 146 groups of soil samples were collected from the Yellow River Basin; 60 samples were collected from the upper reaches of the Yellow River Basin (Helan County, Yinchuan City, and Yesheng Town, Qingtongxia City), and 86 samples were collected from the lower reaches (Lankao County, Kaifeng City). Considering the midstream area, which is influenced by anthropogenic factors such as the construction of terraces and silt dams, sampling in this area was not selected. The upper reaches were sampled over a depth of 0–3 m at 10 cm intervals. The lower reaches were sampled over a depth of 0–5 m. Different textured soils in the study area were divided into four layers, namely, the top soil layer (0–1 m), the second layer (1–2 m), the third layer (2–3 m), and the fourth layer (3–5 m).

The samples collected from the field were dried in an oven and removed small stones, roots, small brick pieces, and other debris. Manual grinding to fine particles, and then through mesh size of 2 mm sieve. The sieved soil sample was placed in a beaker, and 10 mL of 10% H_2_O_2_ solution was added to remove the organic matter from the soil. After the reaction is complete, add 10 mL of 10% HCl solution to the beaker to remove the organic carbon. After filling with distilled water and standing for 12 h, the supernatant was withdrawn. Then, 10 mL of 0.06 mol/L sodium hexametaphosphate solution was added to disperse the soil particles, and after ultrasonic vibration for 15 min, the characteristics of soil particle size distribution were measured by a laser particle size analyzer. To prevent changes in the nature and composition of the samples during drying, the oven used for the experiments was a low-temperature blast drying oven. The soil textures and particle size ranges were determined using a QT-2002 automatic laser particle size analyzer (Channel Science Equipment Co., Ltd., Beijing). The repeatability error was less than 1%. Particle-size volumes and cumulative particle-size volume percentages were measured for 130 particle size intervals in the size range of 0–2000 μm. The data were then analyzed to determine the D of the 356 groups of samples.

**Figure 2:**
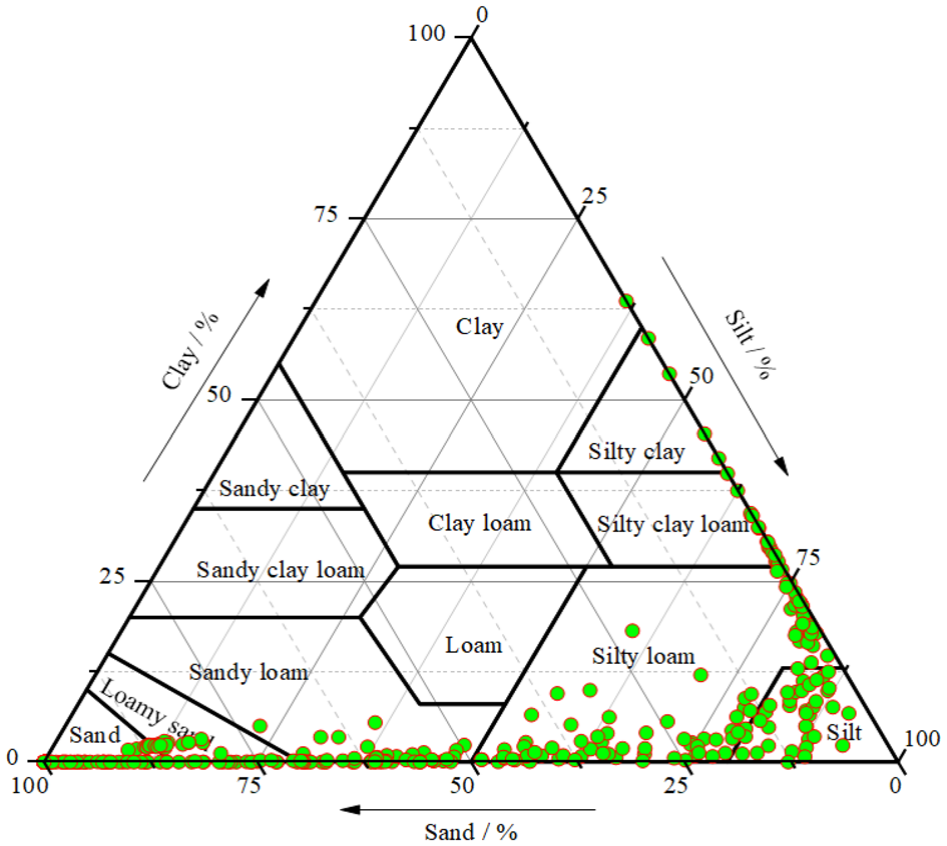
Triangle map of soil texture classification.

According to the American soil texture classification standard, soil particles with diameters of 2–0.02 mm, 0.02–0.002, and < 0.002 mm were classified as sand, silt, and clay, respectively, to derive the values of soil particle composition ratio for the watershed (Table 2). The d_min_ is the minimum value (< 0.02 mm) of the particle size distribution of the soil, which is obtained from the laser particle sizer experiment. d_max_ can also be derived from the particle size distribution data (2–0.02 mm). The particle size of the test soil was plotted in a soil texture triangle (Fig. 2).

**Table 2:** Composition of soil texture particles under different sedimentary environments.

Sedimentary environments	Soil depth/m	Soil particle size distribution (%)			d_av_/μm (< 0.002 mm)	d_min_/μm (2–0.02 mm)	d_max_/μm
		Sand	Silt	Clay			
Upper yellow river	0–3	66.1	33.06	0.84	69.98	0.67	370.88
Lower yellow river	0–3	51.46	39.67	8.87	88.80	0.25	486.08
Mountain-front plain	0–3	57.09	38.34	4.57	48.02	0.43	282.98
Central alluvial plain	0–3	44.18	49.04	6.78	55.88	0.33	370.88
Eastern coastal plain	0–3	32.83	59.43	7.74	55.12	0.33	444.17

### Determination of the fractal dimensions of the soil samples

To calculate the fractal dimension of the soil particle size distribution, it is accurate and simple to use the fractal equation derived from the soil particle size volume distribution data. The fractal dimension can be obtained using the Tyler model^20^. Assuming that the porous soil with a certain self-similar structure is composed of particles with different volumes, the area *A* occupied by soil particles larger than a certain characteristic scale *R* in the two-dimensional plane is^40^:

1 $$A(r > R) = C_{a} [1 - (R/\lambda_{a} )^{2 - D} ]$$

where *r* is the measurement scale (μm); $C_{a}$, $\lambda_{a}$ are constants, are related to the size and shape of the particles. Extending Eq. (1) to three dimensions, the volume *V* of soil particles larger than a certain grain size $R_{i}$($R_{i} > R_{i + 1}$, i = 1, 2, 3, …) is:

2 $$V({\text{r}} > R_{i} ) = C_{V} [1 - (R_{i} /\lambda_{V} )^{3 - D} ]$$

where $C_{V}$ is a constant indicates the sum of the volume of each particle size V0; $\lambda_{V}$ is a constant indicates the maximum particle size $R_{\max }$, and the maximum particle size of the soil in this study was 2,000 μm. So there are:

3 $$V({\text{r}} < R_{i} )/V_{0} = (R_{i} /R_{\max } )^{3 - D}$$

where *D* is the fractal dimension; $V({\text{r}} < R_{i} )$ is the cumulative volume of particles with particle size less than $R_{i}$, as a percentage by volume; $R_{i}$ is the average of the upper and lower particle sizes of the corresponding particle class range. Taking the logarithm of both sides of Eq. (3) simultaneously:

4 $$\lg \left[ {V(r < R_{i} )/V_{0} } \right] = (3 - D)\lg (R_{i} /R_{\max } )$$

In the calculation, the values of $\lg \left[ {V(r < R_{i} )/V_{0} } \right]$ and $\lg (R_{i} /R_{\max } )$ were first determined. Next, the double logarithmic curve was constructed with $\lg \left[ {V(r < R_{i} )/V_{0} } \right]$ as the vertical scale and $\lg (R_{i} /R_{\max } )$ as the horizontal scale. The slope of the line is equal to 3-*D*, which results in the value of the fractal dimension *D*.

## Results and analysis

### Particle size distribution characteristics under different sediment types

#### Characteristics of particle composition

**Figure 3:**
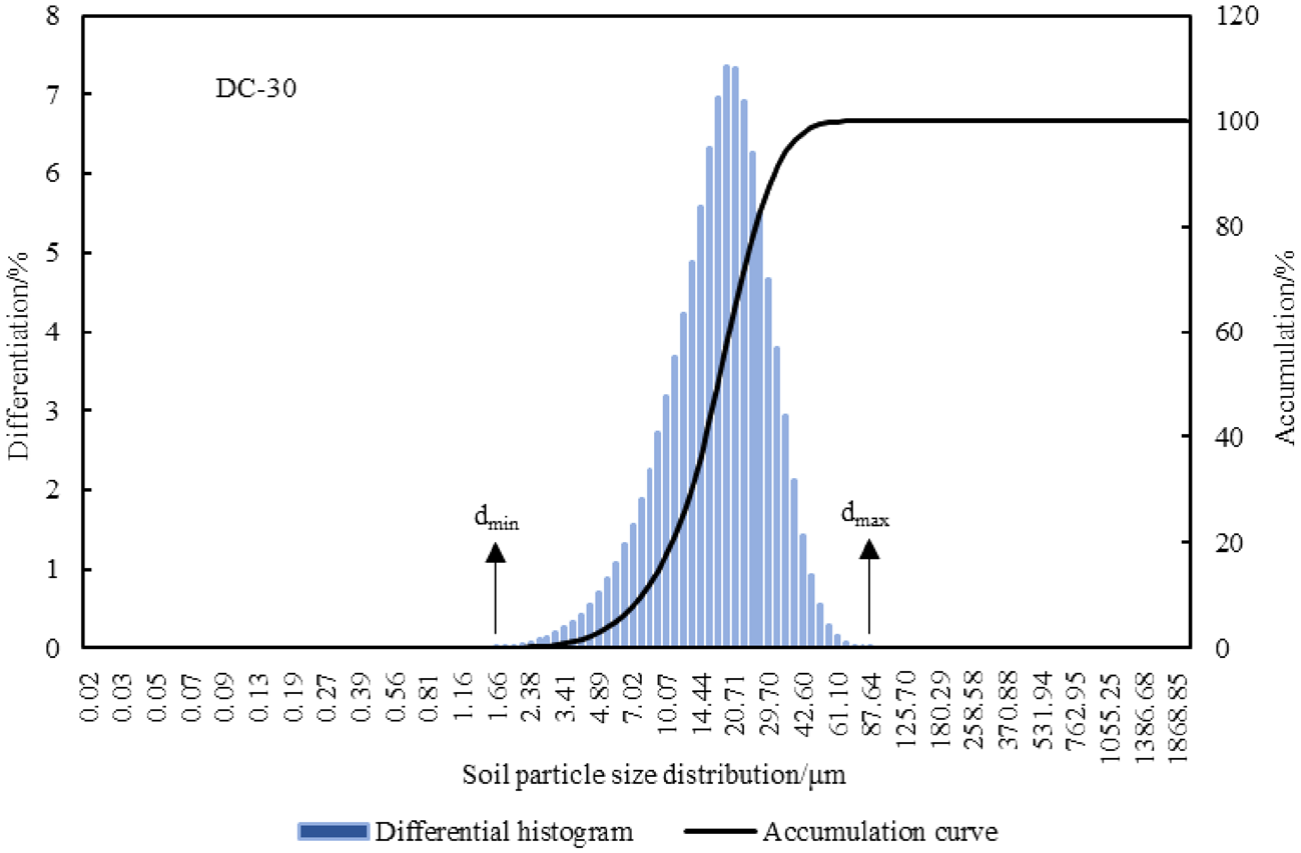
Volume content curve of soil PSD at DC sampling site.

There are many types of soil textures in the typical sedimentary environments of the two basins, and the structural changes are complex. The texture types are mainly sandy soil, loamy sand, sandy loam, silt loam, and silty soil, with small amounts of clay, silt clay loam, and silt clay (Fig. 2). The volume content distribution curve of the particle size distribution of soil particles at a single point is shown in Fig. 3. Among the different geological environments, the sand content was highest in the upper Yellow River area (66.1%) and the lowest in the eastern coastal plain area (32.83%), progressively decreasing from the upper Yellow River area to the mountain-front plain, lower Yellow River area, central plain and then the eastern coastal plain. The silt content showed an opposite trend compared to that of sand content. The clay content in the lower Yellow River area (8.87%) was the highest, followed by the eastern coastal plain (7.74%), the central alluvial plain (6.78%), the upper Yellow River area (4.57%), and then the mountain-front plain (0.84%) (Table 2). According to the values of d_av_, d_min_, and d_max_, it was found that the soil particle sizes in the upper and lower Yellow River areas are more diverse than those in the mountain-front plain, the central alluvial plain, and the eastern coastal plain. Due to river transportation, the sand content decreased from upstream to downstream in the Yellow River Basin, and the silt and clay content increased. The mountain-front area of the Haihe River Basin is affected by the Taihang Mountains, and the soil texture is relatively more uniform. Whereas, the central and coastal areas are affected by alluvial and marine deposits, and the soil has finer grains.

#### Fractal variation characteristics

The fractal dimensions of 356 soil sample groups from the Yellow River and Haihe River Basins were calculated according to the Tyler fractal model. The average D_av_, minimum D_min_, and maximum D_max_ values of the same sedimentary environments were plotted in Table 3. The D values ranged from 0.14–2.03 in the Yellow River Basin and from 0.16 to 2.24 in the Hai River Basin. D_av_ can reflect the general characteristics of the fractals in the study area, and it can be seen that the fractal dimension was the largest in the mountain-front plain at 1.91 and the smallest in the lower Yellow River area at 1.07. In the catchment, the D values were generally greatest for the mountain-front plain, followed by the eastern coastal plain, upper Yellow River, central alluvial plain, and then lower Yellow River, which indicated that the soil particles in the mountain-front plain were denser, while those in the lower Yellow River area were looser. The d_max_ was largest in the lower Yellow River region and the smallest in the mountain-front plain, which is diametrically opposite to the trend in D_av_, indicating that the fractal dimension may be influenced by the large particle size (Table 3).

**Table 3:** Soil fractal characteristics under different sedimentary environments.

Sedimentary environments	Sample size	D_av_	D_min_	D_max_	Standard deviation	Bias angle	Peakedness
Upper yellow river	60	1.28	0.49	2.03	0.40	− 0.35	− 0.72
Lower yellow river	86	1.07	0.14	1.86	0.51	− 0.05	− 1.57
Mountain-front plain	60	1.91	1.47	2.24	0.26	− 0.43	− 1.61
Central alluvial plain	90	1.25	0.18	1.85	0.36	− 1.33	1.42
Eastern coastal plain	60	1.42	0.16	2.19	0.39	− 1.73	3.37

**Figure 4:**
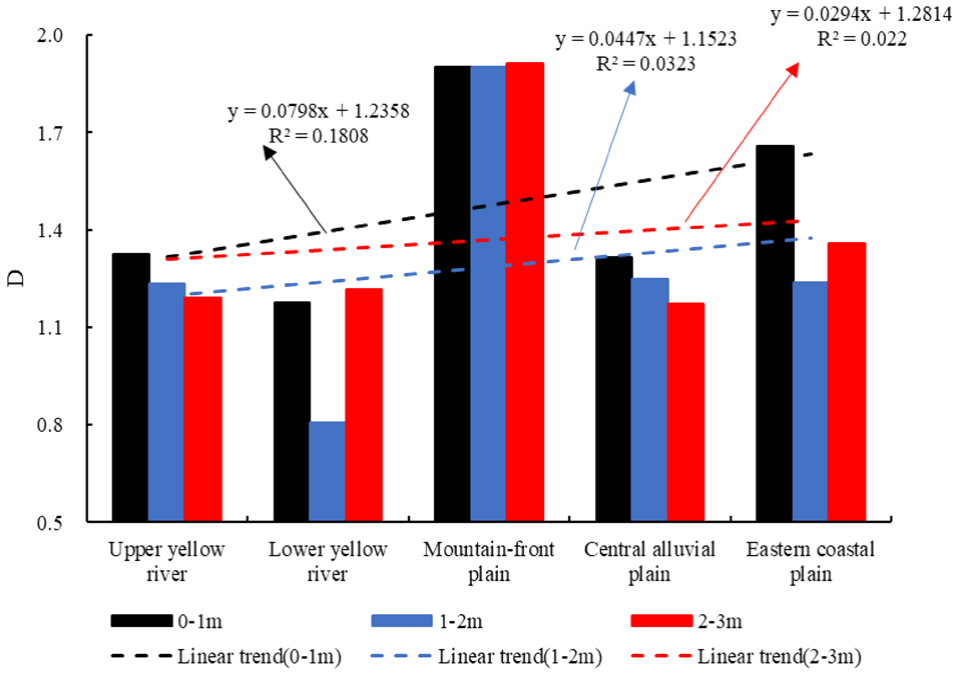
Trend of fractal dimension of the shallow, middle, and deep soils.

**Figure 5:**
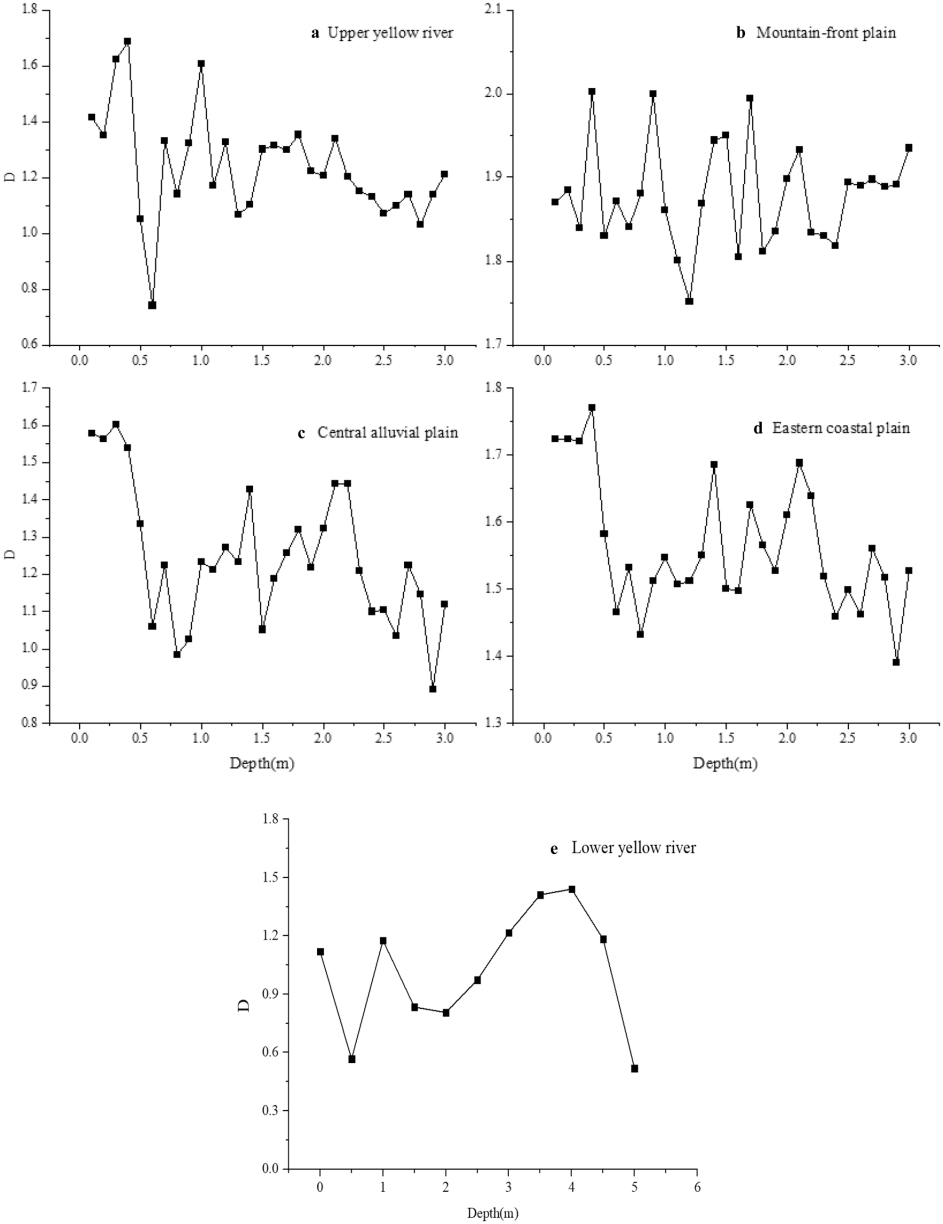
Characteristics of the variation of fractal dimension with depth.

The burial depth of 0–3 m was divided into three layers: shallow (0–1 m), medium (1–2 m), and deep (2–3 m), to characterize the fractal variation at the same depth in different areas (Fig. 4). The ordinate D value in Fig. 3 is the average value of fractal dimension at the same depth in each typical sedimentary environment. It can be seen that the fractal dimension in the upper and lower reaches of the Yellow River is lower than that in the Haihe River Basin plain area, and the fractal dimension of the shallow soil is generally higher than those of the middle and deep soils in the vertical direction. The mean values of the soil fractal dimension at the same depth were integrated, and the curve of D variation with burial depth is plotted in Fig. 5. As can be seen from Fig. 5, the soil fractal dimensions of the basin generally show a decreasing trend with increasing burial depth. In the upper Yellow River area, a significantly small value appears at 0.6 m. In the lower reaches, owing to the limited number of sampled layers, the variation characteristics of the fractal dimensions generally show a decreasing trend with depth. The fractal characteristics of the soils in the central and eastern coastal plains show a trend of gradual decrease with increasing burial depth; the soils in the mountain-front plains present an exception to this trend as their fractal characteristics are stable in the range of 3 m, and the most complex changes are found in the central alluvial plains.

### Correlation of soil properties

#### Correlation of soil physical properties under different sedimentary environments

Based on the Pearson correlation analysis, the correlations of the soil particle size characteristic parameters were obtained for a variety of sedimentary environments (Table 4). The fractal dimension D of soil particles in the upper and lower reaches of the Yellow River Basin was significantly negatively correlated with particle size minimum d_min_ and sand content (P < 0.01), and it was positively correlated with clay and silt content (P < 0.01). There was no significant correlation between D-value and d_max_ in the upper reaches, while the D-value of the lower reaches showed a significant negative correlation with d_max_ (P < 0.01). Both d_max_ and d_min_ showed a significant positive correlation with sand particle content (P < 0.01) and a significant negative correlation with silt and clay particle content (P < 0.01).

**Table 4:** Correlation of soil particle size characteristic parameters under different sedimentary environments.

Sedimentary environments	Indicators	D	Soil	Silt	Clay	d_min_ (< 0.002 mm)	d_max_ (2–0.02 mm)
Upper yellow river	D	1					
	Sand	− 0.655**	1				
	Silt	0.650**	− 0.999**	1			
	Clay	0.541**	− 0.716**	0.683**	1		
	d_min_	− 0.718**	0.640**	− 0.646**	− 0.360*	1	
	d_max_	− 0.148	0.508**	− 0.522**	− 0.132	0.607**	1
Lower yellow river	D	1					
	Sand	− 0.791**	1				
	Silt	0.792**	− 0.957**	1			
	Clay	0.483**	− 0.717**	0.485**	1		
	d_min_	− 0.811**	0.675**	− 0.686**	− 0.388**	1	
	d_max_	− 0.776**	0.831**	− 0.808**	− 0.564**	0.915**	1
Mountain-front plain	D	1					
	Sand	0.778**	1				
	Silt	− 0.816**	− 0.991**	1			
	Clay	− 0.243	− 0.645**	0.538**	1		
	d_min_	− 0.221	− 0.175	0.221	− 0.039	1	
	d_max_	− 0.153	− 0.165	0.201	− 0.019	0.914**	1
Central alluvial plain	D	1					
	Sand	− 0.710**	1				
	Silt	0.751**	− 0.956**	1			
	Clay	0.310**	− 0.688**	0.443**	1		
	d_min_	− 0.834**	0.690**	− 0.713**	− 0.341**	1	
	d_max_	− 0.486**	0.817**	− 0.761**	− 0.609**	0.688**	1
Eastern coastal plain	D	1					
	Sand	− 0.648**	1				
	Silt	0.692**	− 0.972**	1			
	Clay	0.302*	− 0.740**	0.560**	1		
	d_min_	− 0.854**	0.764**	− 0.821**	− 0.343**	1	
	d_max_	− 0.424**	0.785**	− 0.800**	− 0.475**	0.752**	1

In the Haihe River Basin, D is significantly correlated to soil particle content in the central and coastal plain areas. D showed a significant positive correlation with sand content (P < 0.01) and a significant negative correlation with silt and clay content (P < 0.01). The variation in D was also influenced by the bias of large and small particle sizes, and it showed a significant negative correlation with d_min_ and d_max_. Furthermore, the maximum and minimum values of particle size were closely related to the distribution characteristics of soil particles, showing that d_max_ and d_min_ values increased with increasing sand content (P < 0.01). However, there was no significant correlation between the soil fractal dimension and d_min_ and d_max_ in the mountain-front plain area, and D showed a significant positive correlation with sand particle content and a significant negative correlation with silt particle content. Further, there was no significant correlation between d_min_, d_max_, and soil particle content.

#### Correlation of soil properties in different basins

**Figure 6:**
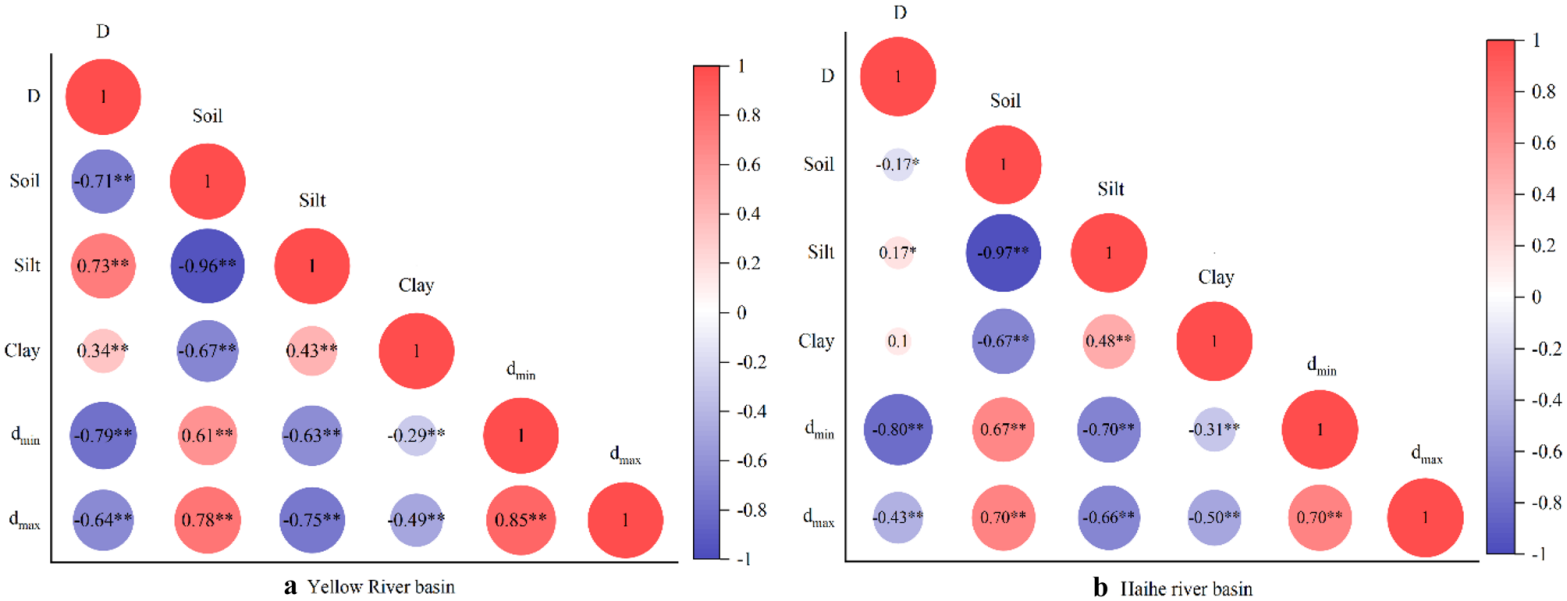
Correlation of soil physical parameters in the Yellow River and Haihe River basins. **Indicates correlation is significant at the 0.01 level; *Indicates correlation is significant at the 0.05 level.

Considering the correlations among the basic soil parameters of all samples from the Yellow River and Hai River basins through different depositional environments, as shown in Fig. 6, the fractal dimension of soils in the Yellow River Basin was significantly negatively correlated (P < 0.01) with sand grain content, d_min_, and d_max_, and it was significantly positively correlated (P < 0.01) with silt particle content and clay particle content. The soil fractal dimension D in the Haihe Basin was significantly negatively correlated with sand content (P < 0.05), significantly positively correlated with silt content (P < 0.05), significantly negatively correlated with d_min_ and d_max_ (P < 0.01), and not correlated with clay content. It can be seen that the fractal dimensions of soil particle size in the Yellow River and Haihe River basins are affected by soil texture and are closely related to the large and small particle size in the sorting and deposition process. The fractal dimension is influenced by soil texture, and the larger the sand content, the larger the soil particle size.

#### Correlation curve

**Figure 7:**
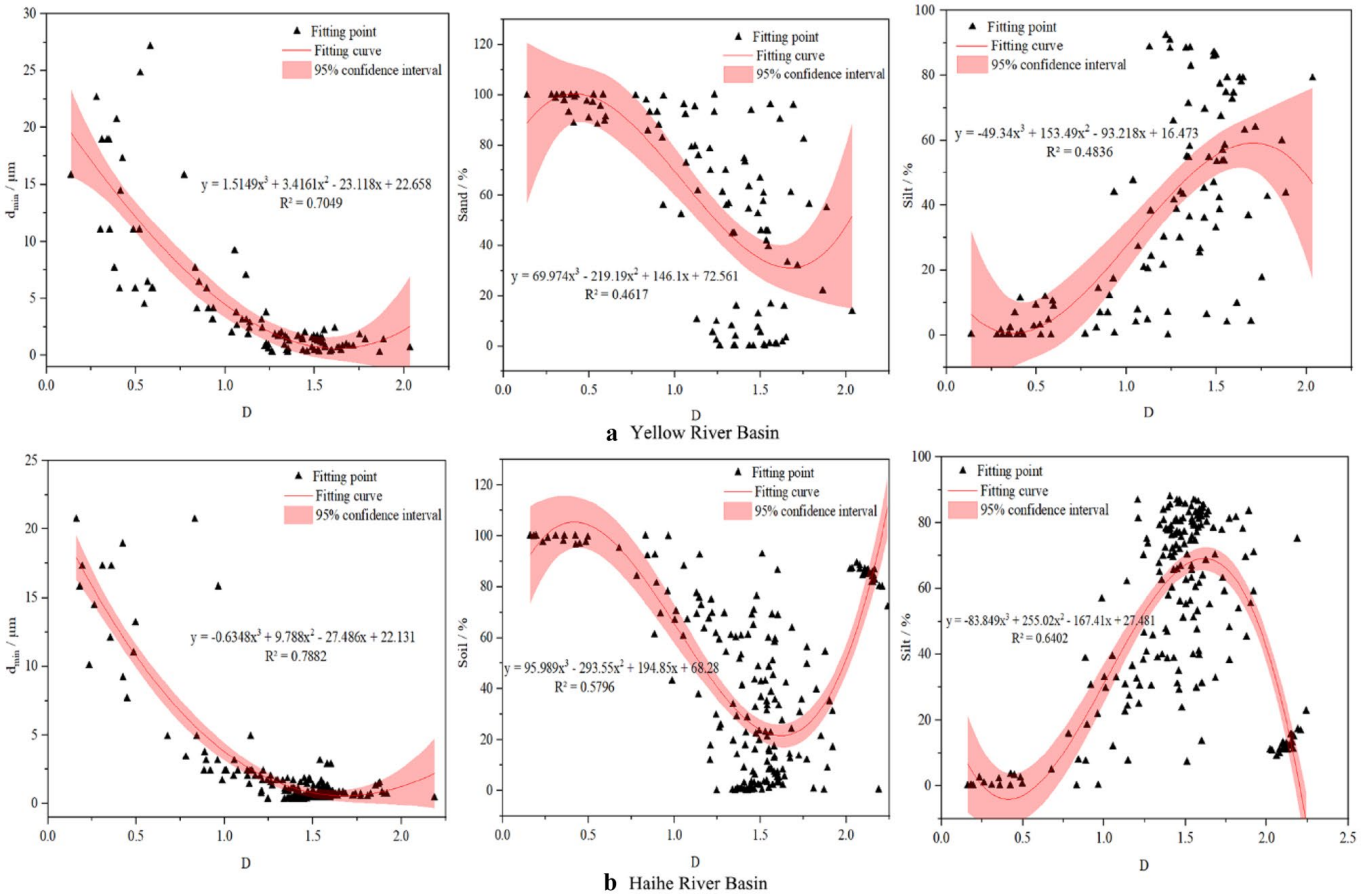
Fitting curves of D with d_min_, sand content, and silt content for individual basins.

Based on the correlation between the fractal dimension of the soil in the basin and the basic parameters of particle size characteristics, the parameters with a higher degree of correlation were selected to establish the fitting curve. Curve estimation using SPSS software yielded a cubic polynomial distribution of the correlation between the soil fractal dimension, particle size characteristics, and particle content. The fitted curves of D versus d_min_, sand content, and silt content were established using the origin and are shown in Fig. 7. The fitting curve of D and d_min_ has a good correlation, showing that the smaller the particle size, the smaller the fractal dimension. This indicates that the fractal dimension is influenced by the abundance of small particles in the particle sorting process under the influence of river alluvial transport, and mountain ranges on precipitation.

The relationship modeled between the fractal dimension of the watershed soil and the grain size minimum (< 0.002 mm) is shown in Eq. (5), which is suitable for two watersheds. The d_min_ is determined by the particle size distribution of soil particles, and the fractal dimension D value is obtained by substituting this equation.

5 $$D = - 0.0002{\text{d}}_{\min }^{3} + 0.0104{\text{d}}_{\min }^{2} - 0.2065{\text{d}}_{\min } + 1.6643_{{^{{_{{}} }} }}^{{}} R^{2} = 0.785$$

This equation requires fewer parameters and provides a simple and quick understanding of the soil particle size distribution characteristics in the different sedimentary environments of the basin.

## Discussion

Soil PSD is used as an aid in distinguishing geomorphology and soil types in areas with different geomorphological units^41^, and it can reflect the variability of different geological environments within a watershed. Guo et al.^42^ found that the particle size composition of sand particles can visually reflect the main particle size composition in wind-formed sand and the relative content of sand particles among different particle size groups. The soil particle composition in their study area has distinctive multiple fractal characteristics. In our study of soil physical parameters in the Yellow River and Haihe River basins, the particle distribution characteristics in the Yellow River Basin showed a general trend of decreasing sand content in the upper reaches with increasing depth. This may be because small particles with a large specific surface area are susceptible to erosion during transport, thus causing environmental problems such as soil erosion in the upper reaches^43^. However, the lower riparian reaches are affected by flow deposition, and the parent material of the soil is carried, sorted, and deposited by the flow of the Yellow River. The content of large particles increased with increasing depth, which is quite different from other regions and is consistent with the conclusions of previous research. Alternatively, Hou et al.^44^ found that the content of sediments with small particle size increased along the river channel in the lower Yellow River. The fine particle content in the Haihe River Basin is higher than that in the Yellow River Basin because the particles and sediments transported by rivers often do not have uniform particle size characteristics. In general, as the transport distance increases, the average diameter of the particles decreases, and the degree of sorting improves^45^. However, the conclusions of this study are inconsistent; in the Haihe River Basin, we found that sediments in the mountain-front area had a smaller average diameter than those in the central and eastern coastal areas, which may be influenced by external factors such as the impact of the Taihang Mountains and the spatial variability of the soil particles.

The fractal dimension D, as a parameter describing the geometry of the soil particles, is closely related to the inhomogeneity of the soil particle size during sorting. Numerous studies have shown that the smaller the soil particle diameter (sand, silt, clay), the larger the fractal dimension of PSD^9,46,47^. In this study, D was significantly positively correlated with soil silt content and significantly negatively correlated with soil sand content and minimum particle size, as analyzed by Pearson correlation results. This study is consistent with the previous studies^49–51^. These results indicate that soil texture has a significant effect on D and is also influenced by the size of the soil particles. By analyzing the relationship between the fractal dimension and particle size characteristics, it can be seen that the fractal dimension of sediments in the mountain-front area in the Haihe Basin is the highest, and the particle dispersion is small. The fractal dimensions of sediments in the central and coastal plain areas are smaller than those of sediments in mountain-front plain areas, and the particle dispersion is relatively poor. This also validates the conclusion that the lower is the fractal dimension, the looser is the soil structure and the poorer is the soil water holding capacity and nutrient retention capacity^48^. However, in the Yellow River Basin, the fractal dimension in the upper reaches with coarser soil particles was greater than that in the lower reaches with finer soil particles. This is not consistent with the conclusion, which may be due to the uneven distribution of soil PSD and the insufficient density of sampling points.

To some extent, D can characterize the homogeneity of soil texture at different soil depths in the basin. From Fig. 6, it was clear that the relationship between the fractal dimension of basin soils and soil PSD was more appropriately described by a cubic polynomial equation. The fitted relationship of the fractal model [Eq. (3)] had a strong correlation and was applied to the different soil layers observed in this study. These results are similar to those of Zhao et al.^52^, demonstrating the validity of using the fractal dimension of PSD as a descriptive parameter for basin soils.

## Conclusions

The soil texture types in the typical sedimentary environments of the Yellow River and Haihe River basins are mainly sand, loamy sand, sandy loam, silty loam, and silty soil, with a small amount of clay, silty clay loam, and silty clay. The soil particle size in the Yellow River Basin was more diverse than that in the Haihe River Basin. The Yellow River showed a trend of decreasing sand content and increasing silt and clay content from upper to lower reaches. While the mountain-front plain areas were subject to the influence of the Taihang Mountains, the soil texture is relatively more uniform than in other sedimentary environments. The central and coastal plain areas were subject to the action of alluvial and marine deposition, and the soil texture is fine-grained.

In the catchment, the D value was greatest in the mountain-front plains, followed by the eastern coastal plain, upper Yellow River, and central alluvial plain, and it was smallest in the lower Yellow River. In the vertical direction, the D value showed a decreasing trend with increasing burial depth, and the fractal dimensions of shallow soils were higher than those of the middle and deep soils. Except for the fractal characteristics of the soil in the mountain-front area, which was less variable within the depth of 3 m from the surface, those of soils in the central and coastal plain areas showed a trend of gradual decrease, and the changes were most complex in the central alluvial plain areas.

The fractal dimension of soil particle size was affected by the soil texture and was closely related to the inhomogeneity of particle size in the sorting processes. However, the fractal dimension of soils in the plains of the Haihe River Basin was less affected by texture, and there was no significant correlation between the fractal dimension and the maximum and minimum grain sizes of soils in the mountain-front areas under the influence of the action of the Taihang Mountains and other factors. The fitted curves of D and d_min_ (< 0.002 mm) correlate well and show a trend of smaller fractal dimensions with smaller particle sizes. The relationship between the fractal dimension and the minimum grain size of the soils in the Yellow River and Hai River basins was modeled as $D = - 0.0002{\text{d}}_{\min }^{3} + 0.0104{\text{d}}_{\min }^{2} - 0.2065{\text{d}}_{\min } + 1.6643$. Using this model, soil particle size distribution characteristics of a river basin can be identified quickly and easily.

However, while the selection of typical samples in this study took into account factors such as regional geomorphological divisions and land use in the study area, the level of diversity of typical samples may not be suitable for all regions. Different terrestrial environments may cause soil differences in terms of parent material, developmental environment, and history. More validation tests are needed in the future to fully investigate the particle size characteristics and homogeneity of soils within a catchment.
